# Diagnosis and management of subarachnoid haemorrhage

**DOI:** 10.1038/s41467-024-46015-2

**Published:** 2024-02-29

**Authors:** Suneesh Thilak, Poppy Brown, Tony Whitehouse, Nandan Gautam, Errin Lawrence, Zubair Ahmed, Tonny Veenith

**Affiliations:** 1 University Hospitals Birmingham NHS Foundation Trust, Queen Elizabeth Hospital, Birmingham, B15 2GW UK; 2 Institute of Inflammation and Ageing, University of Birmingham, Birmingham, B15 2TT UK; 3 Centre for Trauma Sciences Research, University of Birmingham, Birmingham, B15 2TT UK; 4 Department of Critical Care Medicine and Anaesthesia, The Royal Wolverhampton NHS Foundation Trust, New Cross Hospital, Wolverhampton, WV10 0QP UK

**Keywords:** Brain injuries, Neuroscience, Cerebrovascular disorders

## Abstract

Aneurysmal subarachnoid haemorrhage (aSAH) presents a challenge to clinicians because of its multisystem effects. Advancements in computed tomography (CT), endovascular treatments, and neurocritical care have contributed to declining mortality rates. The critical care of aSAH prioritises cerebral perfusion, early aneurysm securement, and the prevention of secondary brain injury and systemic complications. Early interventions to mitigate cardiopulmonary complications, dyselectrolytemia and treatment of culprit aneurysm require a multidisciplinary approach. Standardised neurological assessments, transcranial doppler (TCD), and advanced imaging, along with hypertensive and invasive therapies, are vital in reducing delayed cerebral ischemia and poor outcomes. Health care disparities, particularly in the resource allocation for SAH treatment, affect outcomes significantly, with telemedicine and novel technologies proposed to address this health inequalities. This article underscores the necessity for comprehensive multidisciplinary care and the urgent need for large-scale studies to validate standardised treatment protocols for improved SAH outcomes.

## Abstract (web summary)

Aneurysmal subarachnoid haemorrhage, with its multisystem effects, presents a substantial challenge to clinicians. Here, the authors show the necessity for comprehensive multidisciplinary care and the urgent need for largescale studies to validate standardised treatment protocols for improved outcomes.

## Background

Subarachnoid haemorrhage (SAH), a neurovascular emergency with an incidence in the UK, is approximately 8 per 100,000 population, peaking at 50–60 years and is 1.6 times more common in women than men^1^. The spontaneous rupture of an intracranial aneurysm (80–85%) is the most common cause of SAH^2^. Aneurysmal SAH (aSAH) results in substantial morbidity, mortality and burden on the healthcare system; its downstream effects trigger a cascade of events resulting in organ dysfunction. After initial ictus, these complex ensuing processes cause significant morbidity and mortality. This complex neurovascular syndrome requires an established multidisciplinary team and is best managed in dedicated neurosciences units^3^.

## Anatomy

**Fig. 1:**
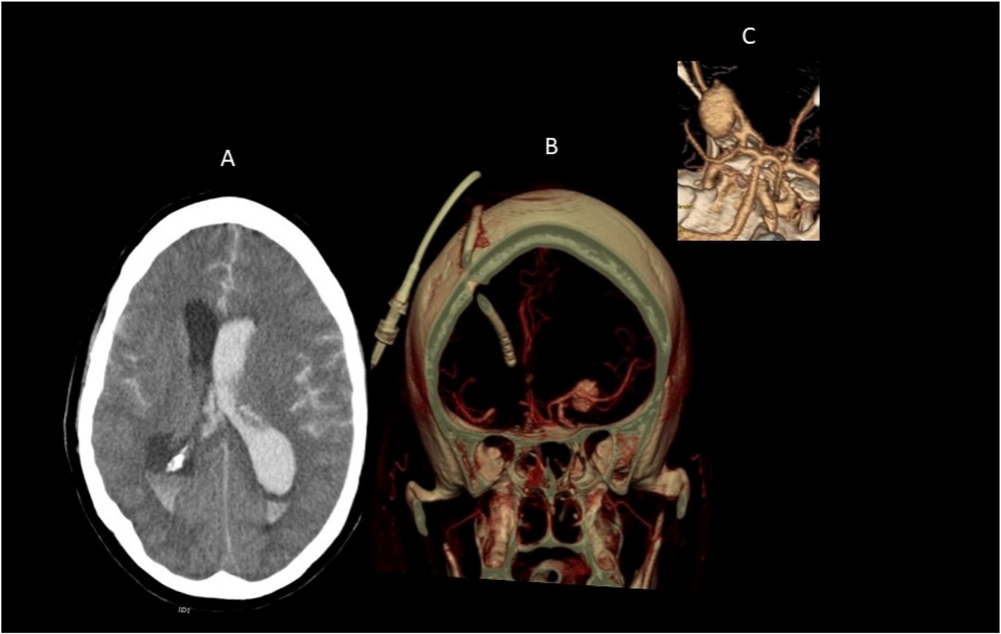
Computerised tomography (CT) of the brain and CT Angiography of subarachnoid and intraventricular haemorrhage. **A** Computerised tomography scan showing subarachnoid and intraventricular haemorrhage; **B** Culprit aneurysm shown on 3D rendering from CT angiography, treated with an external ventricular drain; **C** Closer view of unruptured aneurysm.

Most aneurysms are found in the anterior circulation of the Circle of Willis (Fig. 1). Aneurysms in the posterior circulation of the vertebral and basilar systems are less frequent, accounting for only 12% of intracranial aneurysms^4^. History of familial aneurysms (at least one first-degree relative with an intracranial aneurysm^5^) and certain genetic diseases such as autosomal dominant polycystic kidney disease, Ehlers-Danlos syndrome type IV, Marfan’s syndrome, and neurofibromatosis type 1 have been identified as predisposing factors to cerebral aneurysms^6,7^.

## Prognosis

### Rupture

The incidence of rupture is approximately 0.95% annually. The factors associated with an increased risk of rupture of a cerebral aneurysm are hypertension, smoking, cocaine and alcohol usage. The risk of rupture also varies according to the size (>7 mm) and location, with anterior and posterior aneurysms at a higher risk of rupture than middle cerebral artery (MCA) aneurysms. A daughter sac (irregular protrusion on the sac wall of the aneurysm) and a large size ratio, defined as the aneurysm to vessel size ratio, also increase the risk of aneurysmal rupture^8,9^.

### Outcomes

They vary significantly, from complete recovery to severe disability or death. The most important early predictor of outcome is intact consciousness, with patients having better Glasgow coma score (GCS) at presentation faring better^10,11^: Survival after aSAH has increased by 17% in the past few decades due to early diagnosis, early minimally invasive interventions of aneurysm securement, nimodipine usage and intensive care support^12^. Cumulative case fatality rates after SAH are 25–30% on day one, 40–45% within week one, 50–60% after the first month, and 55–60%, 65% and 65–70% at 6, 12 and 60 months respectively. 12% of patients die before receiving medical attention^13^. Poor outcomes in patients with aSAH are often associated with several key factors. These include older age, worsening neurological conditions, posterior circulation aneurysm rupture, larger aneurysm sizes, and an increased presence of SAH on initial CT scans. Complications such as intracerebral haematoma or intraventricular haemorrhage and elevated admission systolic blood pressure also contribute to unfavourable outcomes. Previous diagnoses of hypertension, myocardial infarction, liver disease, or SAH further compound the risk^14,15^. Intensive care management of aSAH patients primarily focuses on addressing delayed cerebral ischaemia, which affects 20–45% of patients and is linked with worse neurologic outcomes and mortality. However, aSAH also often leads to medical complications like fever, anaemia, and hyperglycaemia, which further influence the patient’s prognosis and hospital stay duration^12,16,17^.

## Diagnosis

SAH should be considered in all patients presenting with sudden-onset severe headaches. Among other presenting features, seizures are observed in approximately 6% of patients^18^. Focal neurological deficits are usually associated with an intraparenchymal haematoma or the location of an aneurysm, e.g. an oculomotor nerve palsy with pupillary dysfunction is associated with enlargement or rupture of the ipsilateral posterior communicating artery aneurysm.

Among patients presenting with an acute nontraumatic headache that has reached maximal intensity within one hour and intact neurology, the Ottawa rules are very sensitive for identifying an SAH but with low specificity^19^. A non-contrast CT scan of the head (CTH), usually modern third-generation CTH, is 100% specific and highly sensitive for aSAH if scanned in the first 6 h of headache onset^20^, the sensitivity decreases to 97% in the first 72 h and further decreases by 50% in 5 days^12^. If CTH performed ≥6 h after the ictus is equivocal or negative, a lumbar puncture performed within 6–12 h of symptom onset typically shows xanthochromia. Unlike CT, xanthochromia is present in the cerebrospinal fluid (CSF) in all patients up to 2 weeks post ictus; false positive results can occur from a traumatic lumbar puncture^21^.

For handling aSAH, computed tomographic and digital subtraction angiography play significant roles^22^. CTA, as a less invasive and readily accessible option, is the first investigation to consider. Apart from identifying aneurysms, it also aids in planning the repair^23^. However, in inconclusive CTA results, Digital Subtraction Angiography (DSA), the gold standard in angiography, is recommended^24^. Furthermore, stereotactic angiography with 3-dimensional rotational capabilities proves valuable for detecting aSAH aneurysms, except when previously diagnosed by a noninvasive angiogram. It contributes to treatment planning and assessing an aneurysm’s suitability for endovascular coiling or surgery^25^. DSA has the added benefit of offering an endovascular treatment and minimal complication rates of 1%. Importantly, studies comparing CTA and DSA indicate strong agreement, suggesting they both provide high sensitivity and specificity in diagnosing vasospasm^26^.

Magnetic resonance angiography (MRA) is an alternative to CTA or DSA for aneurysm detection, especially in patients allergic to iodine. MRA does not expose the patients to radiation, and the time-of-flight sequence does not require contrast. MRA is not very sensitive when done early after symptom onset due to high oxygen concentration in CSF^27^. Magnetic resonance angiogram (MRA) is 95% sensitive for aneurysms >3 mm^28^; the main disadvantages are availability, cost, and acquisition time.

## Pathobiology

Cerebral saccular aneurysms are acquired defects that develop at branch points of major arteries of the circle of Willis. Haemodynamic stress induces degeneration of the internal elastic lamina with thinning and loss of the tunica media, thus forming aneurysms. Aneurysm rupture causes a sudden rise in intracranial pressure (ICP)^29,30^, reducing cerebral perfusion and leading to a transient or persistent ischaemic state^31^. Ongoing ischaemia is associated with mortality and morbidity.

The complex interplay of events resulting in global early brain injury (EBI) such as transient global cerebral ischaemia with diffusion hypoxia due to microthrombosis, Blood-Brain Barrier (BBB) dysfunction, ionic disequilibrium within neurons, and neuroinflammation outside the immediately affected vascular territories makes complete understanding of this phenomenon elusive^32^. The occurrence of cerebral energy utilisation dysfunction, with normal or hyperaemic cerebral blood flow, introduces further difficulties in understanding pathobiology, indicating a possible non-ischaemic mechanism contributing to EBI. This non-ischaemic damage could be linked with widespread cortical spreading depolarisation or mitochondrial dysfunction, leading to aberrant cerebral energy metabolism^33^. Despite these insights, the exact mechanisms of EBI remain uncertain; detailed studies and investigations continue to unfold the reasons for EBI^34^.

In the initial hours after aSAH, the above results in cytotoxic oedema (intraneuronal), progressing to vasogenic oedema (perivascular and extracellular oedema). Cerebral oedema is associated with further inflammation of nervous tissue, excitotoxicity, impaired cerebral autoregulation, microthrombosis, and oxidative stress^35^.

The emerging concept of immune-mediated cerebral damage after aSAH has been clearly defined in experimental and clinical conditions. These immune responses can be assessed using biomarkers like neuron-specific enolase, S100B, high sensitive-CRP, serum procalcitonin, glial fibrillary acid protein and ubiquitin carboxy-terminal hydrolase L1. These biomarkers, used in research and clinical settings, reflect inflammation, neuronal damage, and progression after SAH^36–39^. Once blood enters the subarachnoid space, the neutrophil count increases globally, systemic interleukin 1 (IL-1) and IL-6 levels rise rapidly, whereas IL-10 levels decrease^40^. Neuronal cell death ensues when the neuronal inflammatory processes trigger astrocytes and microglial cells (triggering EBI) in the face of cerebral metabolic distress. This is compounded by sympathetic nervous system activation, cerebral autoregulatory failure, inflammation, and platelet activation, leading to microthrombosis with cortical spreading depolarisation^41^. These factors imply that aSAH could be a systemic inflammatory condition for which innovative therapies might be more impactful than current vasospasm/DCI interventions. Nonetheless, this notion remains under further investigation.

Global cerebral oedema is EBI’s most prominent imaging manifestation, quantified using the subarachnoid haemorrhage early brain oedema score (SEBES). The risk of poor prognosis is doubled in patients with features of EBI. Plasma catecholamines remain elevated for several days post-SAH, and mortality and morbidity are proportional to serum catecholamine concentrations^42^.

DCI usually occurs after three days and is seen for up to 21 days^43^; it is caused by vasospasm, enhanced apoptosis, BBB breakdown, microthrombosis with microcirculatory dysfunction and CSD. DCI was believed to be mainly associated with the narrowing of cerebral arteries beginning days after aSAH, defined as cerebral vasospasm. The CONSCIOUS trial (Clazosentan to Overcome Neurological Ischemia and Infarction Occurring After Subarachnoid Hemorrhage) suggested that vasospasm prevention does not reduce all-cause mortality or DCI. DCI have a multifactorial aetiology related to EBI, arteriolar constriction and thrombosis, cortical spreading ischaemia, and angiographic vasospasm^44^. Over time, the extravasated blood modulates some core factors, such as EBI, resulting in DCI. Overall, the inability of cerebral perfusion to match metabolic demands leads to DCI.

After haemorrhage, free haemoglobin toxicity with transient global ischaemia drives vasoconstriction and neuronal dysfunction^44^. Nitric oxide pathway modulation is a crucial feature of DCI due to decreased production and increased scavenging, linking vascular dysfunction to inflammation and cortical spreading ischaemia. Usually, autoregulatory microvascular dilatation is mediated by astrocyte-derived vasoactive molecules. In aSAH, these mechanisms are inverted, causing vasoconstriction, leading to local hypoperfusion, blood rerouting, and hyperperfusion^45^. These ischaemic and non-ischaemic injuries warrant novel neurotherapies to reduce aSAH-related mortality and morbidity.

## Grading scales

Clinical and radiological grading of subarachnoid and intraventricular haemorrhage is traditionally performed on admission. The preferred scale is the WFNS, which is based on the Glasgow Coma Scale. Severe scores (4 and 5) on this scale indicate a poor outcome. Additionally, the mFisher scale (radiological scale) aids in assessing the extent of bleeding. Other scales such as Apache II, SOFA, and SAPS provide comprehensive health evaluations, while the Hunt and Hess and FOUR scales also find use (Table 1). Newer scales, including SEBES, Hidjra, and VASOGRADE, are now being adopted. These grading systems facilitate multidisciplinary communication and prognostication and identify neurological deterioration early. Factors like patient age, existing health conditions, hyperglycaemia, sepsis, fever, delayed cerebral ischaemia, and rebleeding are also linked with poorer outcomes^10,11,46,47^.

**Table 1:** Grading scales for SAH

Hunt and Hess^5^	Modified Fisher^11^	World Federation of Neurological Surgeons^6^
1: No symptoms or mild headache	0: No SAH or IVH	Grade 1: GCS 15 without focal deficit
2: Moderate to severe headache, stiff neck	1: Focal or diffuse SAH < 1 mm thick, no IVH	Grade 2: GCS 13-14 without focal deficit
3: Drowsy or confused, mild focal neurological deficits	2: Focal or diffuse SAH < 1 mm thick; IVH present	Grade 3: GCSE 13-14 with focal deficit
4: Stupor, hemiparesis	3: Focal or diffuse SAH > 1 mm thick; no IVH	Grade 4: GCS 7-12 with or without focal deficit
5: Coma, decerebrate posturing	4: Focal or diffuse SAH > 1 mm thick; IVH present	Grade 5: GCS < 7 with or without focal deficit

Abbreviations: GCS, Glasgow Coma Scale; IVH, intraventricular haemorrhage; SAH, subarachnoid haemorrhage.

## Management of aSAH

**Fig. 2:**
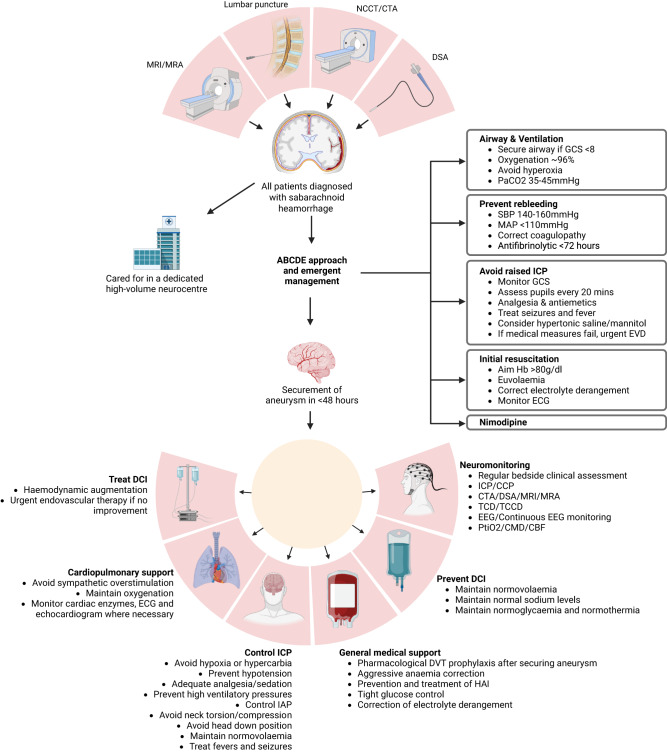
An approach to the management of aneurysmal subarachnoid haemorrhage. NCCT non-contrast CT, CTA CT angiography, DSA digital subtraction angiography, MRI magnetic resonance imaging, MRA magnetic resonance angiography, ABCDE airway breathing circulation disability exposure, GCS Glasgow coma scale, SBP systolic blood pressure, MAP mean arterial blood pressure, ICP intracranial pressure, EVD external ventricular drain, Hb haemoglobin, ECG electrocardiogram, DCI delayed cerebral ischaemia, CPP cerebral perfusion pressure, TCD transcranial doppler, TCCD transcranial colour doppler, EEG elecroencephalogramPtiO_2_-brain tissue oxygenation, CMD cerebral microdialysis, CBF cerebral blood flow, ARDS acute respiratory distress syndrome, IAP intraabdominal pressure, DVT deep vein thrombosis, HAI hospital-acquired infection, LOC loss of consciousness. Created with Biorender.com.

Critical care management of patients with SAH is challenging and requires awareness of all potential medical and neurological complications, with timely intervention and treatment. Care of patients with aSAH requires a multidisciplinary team approach. Outcomes are improved at high-volume centres (institutions that care for at least 35 patients with SAH annually) with dedicated neurocritical care units and specialised team members^3,48^. Aims of critical care management summarised in (Fig. 2) include initial stabilisation of the patient to prevent rebleeding and allow for definite early treatment, limiting secondary neurological injury and early recognition and treatment of complications^49,50^.

## Management before securing the aneurysm

The initial treatment is the same as any critically ill patient: concurrent resuscitation and management of acute neurological decline, avoiding hypoxia and hyperoxia, if airway compromise, GCS < 8 or drop in GCS > 2 points, secure airway. Avoid hypotension, monitor for arrhythmia, avoid hypotonic fluids, maintain normovolaemia, and treat seizures and fever. Initiate neuromonitoring, including pupillary size, shape and reactivity to light every 20 min once the patient is sedated and paralysed.

In patients with aSAH, rebleeding carries very high morbidity and mortality^51^. Rates of aneurysmal rebleeding have drastically improved since the 1980s, when >35% of patients rebled before securing the aneurysm^52^. Currently, 10–15 % of aSAH rebleed, with 50% occurring in the first 6 h^53,54^.The risk factors associated with rebleeding are higher grade aneurysms, hypertension, lower GCS, larger or irregular aneurysms, and delayed securing^53^.

Guidelines recommend reducing blood pressure; mean arterial pressure (MAP) > 95 mmHg may be detrimental^55–57^. The European Stroke Organisation guidelines recommend treatment for BP if systolic BP > 180 mmHg, adjusting the MAP with analgesics, nimodipine and titratable antihypertensives as necessary. The American Heart Association/American Stroke Association (AHA/ASA) guidelines recommend treatment with a titratable agent^50^. However, they suggest a lower systolic BP threshold of <160 mmHg. After decades of research, we still need better evidence on BP targets. Give nimodipine as early as possible to all patients with aSAH; it is a Class I recommendation with level A evidence as per AHA/ASA. Avoid aspirin and other non-steroidal anti-inflammatory drugs (NSAIDs) before securing the aneurysm.

## Management of aneurysm

Definitive treatment hinges on early securement of the aneurysm, within 72 h or ideally within 48 h of diagnosis^58,59^. Most rebleeding happens within the first 24 h^60^, so early securement (within 24 h) may be safe^61,62^. The securement method depends on many factors, particularly the patient’s age, morphology and location of the aneurysm, and presence of intraparenchymal haemorrhage. Following the international subarachnoid aneurysm trial (ISAT), coiling is preferred as it is associated with improved mortality and functional outcomes, with a marginally higher rate of aneurysm recurrence^63–65^. The choice of securement method should be made after a multidisciplinary team discussion between the interventional radiologists, neurosurgeons, and neurocritical specialists. Tranexamic acid may reduce rebleeding and improve overall mortality^66–68^. Current guidelines recommend a short course of antifibrinolytic agent and discontinuing after securing the aneurysm, a maximum of 72 h of tranexamic acid. Prolonged use of antifibrinolytics increases the risk of thrombotic events and DCI^67,69^.

## Management after securing the aneurysm

### Early brain injury

**Fig. 3:**
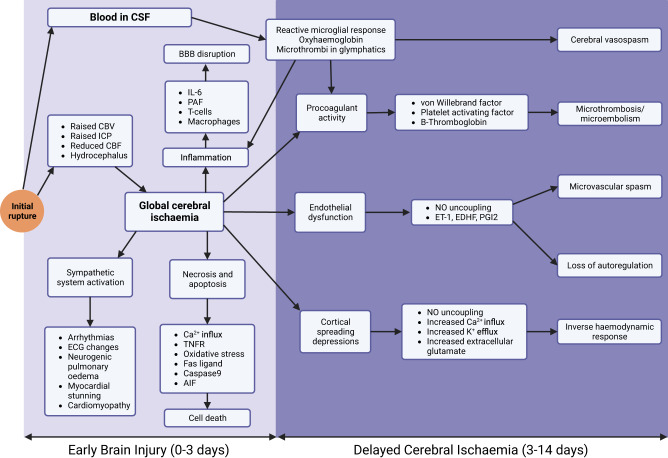
Pathophysiology of aSAH. The time ranges at bottom depict approximate/presumed periods when the various processes occur. CSF cerebrospinal fluid, CBF cerebral blood flow, CBV cerebral blood volume, ICP intracranial pressure, Oxy-Hb oxyhaemoglobin, EDHF endothelial derived hyperpolarising factor, ET-1 endothelin 1, FasL Fas ligand, AIF apoptosis inducing factor, IL-6 interleukin 6, NO nitric oxide, PAF platelet-activating factor, PGI2 prostacyclin, TNFR tumour necrosis factor receptor, vWF von Willebrand factor, DCI delayed cerebral ischaemia, BBB blood brain barrier.

EBI (Fig. 3) occurs within the first 72 h, has a long-lasting impact, and is associated with the development of DCI^70,71^. It increases mortality and morbidity. Those at the highest risk include patients with high-grade haemorrhage, large intracranial blood volume, and prolonged loss of consciousness. Early CT features of brain oedema and ischaemia evidence EBI. Early brain hypoperfusion plays a crucial role in the genesis of EBI^71,72^. There are no current therapeutic modalities to treat established EBI.

Early management of aSAH should focus on preventing the development of EBI by providing adequate brain oxygen delivery and meeting the brain’s metabolic demands. Measures include early securement of aneurysm, avoiding considerable BP changes, treating hydrocephalus, seizures, and cardiopulmonary complications. Optimise cerebral perfusion pressure (CPP) to target >70 mmHg but <95 mmHg early in the treatment, as recent data suggest improved outcomes by improving perfusion and reducing brain metabolic stress. Aggressively treat anaemia to maintain adequate oxygen delivery and control fever to reduce metabolic demand on the brain^34,58^. Advanced multimodality monitoring may assist in individualising treatment strategies. Experimental studies involving agents targeting neuroinflammation like NSAIDs, thromboxane synthase inhibitors, steroids, nitric oxide, and various immunosuppressants have failed to demonstrate benefit. Agents like ketamine targeting cortical spreading depolarisations are under evaluation^72,73^.

### Hydrocephalus

This complication develops within 72 h of aSAH, characterised by ventricular enlargement. Incidence is 20-30% of patients with aSAH. Hydrocephalus can dramatically affect the level of consciousness with improved drainage via CSF diversion (e.g. with an external ventricular drain or lumbar drain).

### Seizures

These are also early complications after an aSAH. Most seizures occur within the first 24 h and may indicate a rebleed^74^. Seizures are common in young patients with MCA aneurysms and the presence of intraparenchymal lesions (e.g. arteriovenous malformations, bleeds or infarcts). Nonconvulsive status epilepticus may be present in up to 20% of patients, especially those with a high-grade haemorrhage or comatose^75^. Antiepileptics are associated with poor cognitive outcomes and increased hospital complications, so routine prophylaxis is not recommended^76–79^. Patients with poor-grade aneurysms and depressed conscious states without clear explanation should have continuous electroencephalograms to look for subclinical seizures and nonconvulsive status epilepticus^80,81^.

## DCI and vasospasm

Development of vasospasm and DCI (Fig. 3) commonly occurs 3–14 days post-aSAH. DCI with cerebral infarction is the leading cause of morbidity in survivors. DCI was purely attributed to vasospasm in the past, but there is radiological evidence of vasospasm in 70% of patients with aSAH without any focal neurology. Recent evidence suggests a complex interplay of pathologies resulting in the development of EBI followed by a DCI^82^. DCI is characterised by any neurological deterioration (focal deficit or decline in GCS by ≥2 points), lasting more than one hour and no other cause. It is reported to occur in about 30% of aSAH patients and is a significant cause of mortality and disability^83^.

Effective treatment of vasospasm does not correlate with decreased incidence of DCI or improved outcomes, as seen in the Clazosentan trials^84^. Also, nimodipine does not affect vasospasm yet improves DCI and patient outcomes^85,86^. Other clinical trials aimed at vasospasm, including magnesium (MASH trial and subsequent meta-analyses)^87,88^, statins^89^ and methylprednisolone^90^, have failed to show outcome benefits. These negative trials reinforce our understanding of DCI’s complex and multifactorial pathophysiology.

Vasospasm development gives an insight into those at high risk for developing DCI. Transcranial Doppler (TCD) and Transcranial Colour Doppler (TCCD) have revolutionised the detection of vasospasm and are widely used at the bedside. It is a noninvasive tool for identifying vasospasm^91^. It has a Class IIa Level of Evidence B recommendation to monitor the development of vasospasm^58^. TCD evidence of vasospasm has a high prediction for the development of DCI. It has a sensitivity of 90% (95% CI 77–96%), specificity of 71% (95% CI 51–84%), Positive predictive value (PPV) of 57% (95% CI 38–71%), and negative predictive value (NPV) of 92% (95%, CI 83–96%)^92^. Pitfalls include availability and operator-dependent variability. TCD is most sensitive to MCA vasospasm. Velocities >120 cm/s in the MCA have a high negative predictive value, and velocities >180 cm/s have a high positive predictive value for the presence of vasospasm. Lindegaard ratio (LR) is the MCA’s mean velocity divided by the ipsilateral extracranial internal carotid artery (ICA). This ratio is usually <3. LR is used to distinguish whether the MCA velocity is secondary to hyperaemia (can also result in flow velocities >120 cm/s) or due to vasospasm. LR < 3 suggests hyperaemia, 3–6 indicates mild vasospasm, >6 severe vasospasm. Serial examinations and trends in velocity are more critical than a single reading. TCD still lacks good evidence for its routine usage outside research^49^.

If symptomatic vasospasm is suspected, immediate CT angiogram (CTA) and CT perfusion (CTP) may be indicated. CTA has become more widely used as it is highly sensitive and specific^93,94^. CTP is emerging as a potential test for hypoperfusion in the presence or absence of large vessel vasospasm. CTP may be able to identify DCI when it is still reversible^95^. Digital subtraction angiogram (DSA) remains the gold standard for vasospasm identification and should be performed if clinical large vessel vasospasm is suspected. This has the advantage of being a diagnostic tool, and treatment can be provided in the same session.

## Multimodality monitoring

Multimodality monitoring in aSAH patients offers a comprehensive approach to assessing the risk of delayed cerebral ischemia. Brain tissue oxygen (PbtO2) monitoring measures invasive partial pressure of oxygen, with normal levels below 20 mmHg indicating risk of ischemia^96,97^. Decreasing levels below 15 mmHg warrants immediate measures to enhance cerebral tissue oxygenation. Interstitial glucose, glycerol, lactate, pyruvate, glutamate, and numerous inflammatory biomarkers constitute markers for cerebral microdialysis (CMD), another monitoring tool. A heightened lactate-pyruvate ratio (LPR) signals anaerobic metabolism, suggesting an essential role in initiating DCI. Information on systemic haemodynamics, vital for managing critically unstable aSAH patients in the ICU, is obtainable from the cardiac output and index. Noninvasive brain oxygenation monitoring, crucial for the early identification of hypoperfusion linked to DCI, is delivered through near-infrared spectroscopy. The transcranial doppler ultrasonography monitors cerebral blood flow velocity and anticipates vasospasm, a common DCI complication. Employing dual or multichannel systems enables simultaneous extensive monitoring, supporting timely intervention and management to impede DCI progression.

## Nimodipine and other therapeutic interventions for DCI in aSAH patients

Currently, nimodipine is the only proven standard neurotherapeutic regimen to prevent and treat cerebral vasospasm and DCI. Mechanisms of action are likely more complex than simple inhibition of vasoconstriction and include reduction of vasospasm, neuroprotection, enhancement of fibrinolytic activity and thus reduction of microthrombosis, and diminishment of cortical spreading depolarisations^98,99^. Systemic arterial hypotension remains the most significant adverse effect of nimodipine treatment^100^. Nimodipine in hemodynamically unstable patients will increase the therapeutic intensity levels, such as using vasopressors, fluids, or frequent reduction and adjustment of its dosage. Nimodipine should be prescribed to all aSAH patients for up to 21 days^101^. The available therapies for DCI include induced hypertension and endovascular treatment. Hypertension is the only “Triple H” therapy component that effectively increases perfusion and brain oxygenation^102,103^. Both hypervolaemia and haemodilution lead to more deleterious effects^104–106^. Blood pressure should be titrated, weighing the risks and benefits stepwise until clinical symptoms improve. If the neurologic deficit persists despite hypertensive therapy, consider endovascular therapy to improve long-term outcomes^107^. Intravascular vasodilators (milrinone, verapamil, nicardipine) are associated with significant improvement in angiographic spasm and neurological signs, but phase three studies are required before their routine use. Angioplasty can be considered if the spasm is refractory to hypertensive therapy. However, prophylactic use of angioplasty is not associated with improved clinical outcomes and may be related to increased risk of arterial rupture; it is not currently recommended^108^.

## Medical complications

Medical complications are common in severe aSAH, affecting the outcomes. Among the prevalent complications are infections such as central line infections, ventilator-associated pneumonia, and deep venous thrombosis. Both prevention and keen surveillance are crucial, along with precise treatment plans to manage these complications. Hospital-acquired infections have been linked to nutritional deficiencies, specifically low glutamine levels, and adverse outcomes^109,110^. Fever increases brain metabolism and exacerbates brain hypoxia in aSAH patients. Therefore, fever prevention/treatment is necessary to reduce secondary brain injury and improve outcomes. Pharmacological antipyretics may not suffice, necessitating additional strategies for maintaining normothermia. It’s essential in critical care to maintain normoglycemia, avoiding aggressive insulin strategies that could cause intracerebral hypoglycaemia in SAH^111^.

Anaemia is also associated with poor outcomes in aSAH^112^. Optimal haemoglobin targets remain controversial and warrant further research. Optimal fluid resuscitation strategies are unclear; hyper and hypovolaemia are deleterious, so euvolaemia is advocated^113^. In the context of resuscitation strategies, maintaining optimal oxygenation is key; hypoxia and hyperoxia should be prevented^114^. Initiation of pharmacological DVT prophylaxis within 24 h of securing the aneurysm is recommended, though controversy persists^115,116^.

Early mobilisation strategies benefit all patients with aSAH, including those with EVDs^117^. The timing for procedures like tracheostomy and percutaneous endoscopic gastrostomy is debatable. In a small retrospective case series, there was no difference in 6-month functional outcomes for early versus delayed tracheostomy (≥10 days) following ischaemic stroke, intracerebral haemorrhage, or aSAH^118^.

Fever is a common association in patients with aSAH, linked to worse outcomes. Central fevers can occur with or without infections, leading to potentially unnecessary antibiotic treatment. Fever prevention and treatment reduces secondary brain injury and improves outcomes^119^ as fever increases brain metabolism and causes brain hypoxia, exacerbating DCI in patients with aSAH. Pharmacological antipyretics might not be sufficient, so adjunctive strategies for normothermia might be necessary.

## Cardiopulmonary

Myocardial injury is a common occurrence following subarachnoid haemorrhage, with initial symptoms including troponin elevations in 28% of patients within the first 24 h^120^, arrhythmias in 35%^121^, and wall motion abnormalities in 28%^122^. Deep septal T-wave inversions, cerebral T waves, and QTC prolongation associated with insular injury are observed on ECG. Nearly all patients experience some ECG abnormality, with severity proportional to the cerebral insult^123^, although findings can vary significantly. Life-threatening arrhythmias, however, occur in only 5% of cases^121^. The degree of troponin increase correlates with the severity of intracranial injury^124^, left ventricular dysfunction, and mortality^125,126^.

Neurogenic stress cardiomyopathy typically requires both transient and reversible left ventricular dysfunction, and a negative coronary angiogram for diagnosis. Retrospective studies estimate incidence among aSAH patients at 1–5%^127,128^.This is due to acute brain-heart interactions following severe brain injury^129^. The pathophysiology of neurogenic cardiac injury is complicated^130,131^, encompassing autonomic dysregulation, excessive catecholamine release, myocyte injury, mitochondrial dysfunction, and ongoing inflammation^132^.

Catecholamine release and sympathetic overstimulation lead to cardiopulmonary dysfunction, which can manifest as subendocardial ischaemia, stunned myocardium, atrial and ventricular arrhythmias and ECG changes (T-wave inversion, ST depressions, or even ST elevations) in the absence of structural coronary artery disease. Echocardiography may show regional wall motion abnormalities, including non-ischaemic cardiomyopathies, such as takotsubo cardiomyopathy with apical ballooning^133^. Cardiac monitoring with electrocardiogram, cardiac enzymes, and echocardiogram is essential in aSAH when there is an insufficient response to ionotropic support during induced hypertension. The development of acute heart failure and hypotension are associated with poor outcomes. SAH patients with symptomatic vasospasm with high initial troponin I level, indicative of neurogenic cardiac injury, are at twice the risk of medical treatment failure^107^. Acute respiratory distress syndrome, and neurogenic and cardiogenic pulmonary oedema have also been described in patients with aSAH. Hypoxia and hypotension harm the brain, contributing to secondary brain injuries. We aim to maintain brain perfusion and oxygenation while treating cardiopulmonary complications.

## Hyponatraemia

Hyponatraemia is commonly seen in association with aSAH. Hyponatraemia (serum sodium of <135 mmol/L) affects 27–44% of patients after aSAH, most commonly from the syndrome of inappropriate antidiuretic hormone (SIADH) or cerebral salt wasting (CSW). CSW is a debated entity amongst clinicians, with some studies suggesting it doesn’t exist as an aetiology for hyponatraemia^134^. Hypovolaemia, hyponatraemia or subsequent fluid restriction increase the risk of DCI with poor outcomes. Therefore, in clinical practice, hyponatraemia associated with SAH management is based on maintaining euvolaemia, avoiding hypo- and hypervolaemia and repletion of volume and sodium losses^135^.

## Managing non-culprit (unruptured) aneurysms

A multidisciplinary team comprising an interventional neuroradiologist and a neurosurgeon should discuss the options, including endovascular coiling, neurosurgical clipping, or conservative management. Follow-up monitoring considers factors including aneurysm size, lifetime risk of rupture, risk of treatment options, and comorbidities. These should be discussed with the patient and their family where appropriate^49^.

## Management of SAH in young people

It is an uncommon disease in children and young adults, with a male-to-female ratio of 1:8 ratio^136^. Anatomically, most of the aneurysms are located at the tip of the internal carotid artery, followed by the posterior circulation, with 60% presenting as an SAH. 30% of SAH in the paediatric population is due to a structural abnormality of the blood vessel wall abnormalities, such as Marfan syndrome, Ehler-Danlos syndrome type IV, fibromuscular dysplasia or arterio-venous malformations. These patients should all be investigated for connective tissue disorders as a part of their work to manage the SAH.

## SAH and inequality of healthcare accessibility

Inequalities within the health care systems, limited access to experienced neurosciences units and lack of skilled staff to manage the SAH within and between countries significantly impact the SAH outcomes. The first step to reducing these inequalities requires access to good-quality data and acknowledging these disparities^137^. An example of such discrepancy is the differences in outcomes of high-volume versus low-volume centres for SAH management. Telemedicine can be a valuable tool that can be leveraged in resource-limited or low-volume settings, providing equitable access to care and expert intervention^138^. Greater attention to community-based educational initiatives to improve stroke awareness and timely intervention enhances patient outcomes^139^. Management of SAH requires urgent large-scale phase three studies to establish standardised protocols and multidisciplinary neurovascular training programmes. The evolution of technology use in resource-poor settings, such as machine learning and artificial intelligence, can advance health equity in aSAH management by predicting disparities and aiding diagnosis and treatment decisions^140^. Techniques, such as novel robotic transcranial Doppler monitoring, may mitigate the requirement for trained staff to manage these patients^141^. Once established, adherence to evidence-based guidelines should be encouraged by data-driven performance analysis^142–144^. Reduction of long-term morbidity after SAH, such as neurocognitive and social decline, requires a close integration of health and welfare services^145–147^.

## Discussion

While advancements in medical technology and treatment protocols have significantly improved outcomes, SAH remains a challenging condition with profound implications for patient care. The highly individualised nature of SAH, due to factors such as aneurysm location patient comorbidities, and pathophysiology underscores the importance of personalised treatment strategies. The incidence of multi-system complications further exemplifies the need for an interdisciplinary approach, which is critical for optimising patient outcomes.

As we continue to face these multifaceted challenges, there is a pressing need for ongoing research to refine existing treatment protocols and to develop new, innovative approaches to manage SAH. Future studies should aim to address the gaps in our understanding of SAH pathophysiology and to evaluate strategies that could mitigate the systemic inequities that currently limit the accessibility of optimal care.

The collaboration between neuroscientists, clinicians, and health policy experts will be crucial in this endeavour. The development of novel therapeutics and improved management techniques, backed by robust data collection and analysis, holds the promise of enhancing the quality of life for individuals affected by SAH.
